# Acknowledgment to Reviewers of *Clinics and Practice* in 2021

**DOI:** 10.3390/clinpract12010011

**Published:** 2022-01-29

**Authors:** Clinics and Practice Editorial Office

**Affiliations:** MDPI AG, St. Alban-Anlage 66, 4052 Basel, Switzerland; clinpract@mdpi.com

Rigorous peer-reviews are the basis of high-quality academic publishing. Thanks to the great efforts of our reviewers, *Clinics and Practice* was able to maintain its standards for the high quality of its published papers. Thanks to the contribution of our reviewers, in 2021, the median time to first decision was 24 days and the median time to publication was 57 days. The editors would like to extend their gratitude and recognition to the following reviewers for their precious time and dedication, regardless of whether the papers they reviewed were finally published:
Abeykoon, JithmaMawhinney, ThomasAbruzzese, ElisabettaMenga, Luca SalvatoreAhluwalia, PankajMesquita, JoanaAkingboye, AkinfemiMessias, AnaÁlvaro-Afonso, Francisco JavierMeštrović, TomislavAnand, SachitMiller, ElzbietaAugustine, JosyMirna, MoritzBalieva, Flora N.Mizuno, KentaroBarausse, CarloMoktefi, AnissaBavaro, DavideMolinari, AngeloBellasi, AntonioMonni, GiovanniBennardo, LuigiMurakhovskaya, IrinaBernabeu-Wittel, MáximoMyakala, KomuraiahBevc, SebastjanNaselli, AngeloBhandarkar, Nikhil SureshNiebel, DennisBonanad, SantiagoNorcic, GregorBorghesi, AndreaOselin, KerstiBoussios, StergiosPadervinskienė, LinaBrembilla-Perrot, BéatricePădureanu, VladBrunnström, HansPaoletti, Anna MariaBucci, TommasoPaul, NicolasButler, Daniel S.C.Pereira, Sofia S.Capocasale, GiorgiaPérez-Herrero, María A.Carugno, JosePogorelić, ZenonChen, Sheng-HungPoluzzi, ElisabettaChoy, RichardPuci, MariangelaCiriaco, PaolaPurrucker, JanContaldo, MariaQueudeville, ManonCortegiani, AndreaRamatchandirin, BalamuruganCrudele, LucillaRazvan-Cosmin, PetcaDay, Andrew S.Recsan, ZsuzsannaDelgado, Bruno MiguelRezus, CiprianDelmonico, LucasRich, PhoebeDervenis, PanagiotisRiquelme-Gallego, BlancaDoll, DietrichRolla, GiovanniDovnik, AndrazRomero, Eleuterio SanchezDuś-Ilnicka, IrenaRoshandel, DanialEblimit, AidenRuszkowska-Ciastek, BarbaraEhrenpreis, Eli D.Saisho, YoshifumiFareed, Muhammad AmberSaito, JumpeiFeo, ClaudioSakamoto, YuzuruFerrara, GiovannaSamujh, RamFiore, Adolfo DiSanti-Cano, María JoséFlora, GaganSantinha, GonçaloFrigy, AttilaSanz-Nogués, ClaraFujioka, KazumichiSapkota, SujataFurfaro, FedericaSarno, LauraGaletta, DomenicoSarria-Santamera, AntonioGarcía-Zamalloa, AlbertoSato, ShuzoGong, WenpingSavarino, VincenzoGooty, Vasu D.Scappaticcio, LorenzoGujski, MariuszSchiavone, MarcoGupta, YashSellmann, TimurHaneke, EckartSerio, GabriellaHirono, KeiichiSerra, RaffaeleHo, PrahladSeverino, PaoloHong, Ji YoungShankargouda, PatilIrani, MehraboonShen, JinleiIriuchishima, HironoShepelkova, GalinaJabłońska, BeataShi, Hon-YiJankowski, MateuszSimurda, TomasJansåker, FilipSocha, Maciej W.Jarosz-Chobot, PrzemysławaStupak, AleksandraJukić, MiroSumin, Alexei N.Kaesmann, LukasSzandruk, MartaKamiya, TakeshiSzmajda-Krygier, DagmaraKanamoto, TaiseiTakahashi, KoichiroKantar, Rami S.Talar-Wojnarowska, RenataKato, HideoTanoubi, IssamKaushik, ShubhiTardáguila-García, AroaKavecan, IvanaTejedor, Félix MarcosKim, Hei-sungTinelli, AndreaKim, Won YoungTrama, FrancescoKoestenberger, MartinTrâmbițaș, CristianKole, ChristoTsai, Han-MouKrebs, Thomas FranzTzang, Bor-ShowKrzymińska-Siemaszko, RomaUpadhyaya, JasbirKwong, AvaUrek, Miranda MuhvicLadani, AmitVasconcelos, CarlosLecka, IzabellaVenugopal, NiranjanLee, Byoung-HeeVerma, NupurLee, SeunghoVillafañe, Jorge HugoLee, Teng-YuVittori, AlessandroLeoni, Matteo Luigi GiuseppeVyas, PriyankaLeyva, Alejandro MolinaWada, HideoLiao, ZehuanWang, ShaobinLiotti, AntoniettaWang, Tzu-ChuehLipinski, PatrykWatanabe, YutakaLopez-Jornet, PiaWilliams, Owen MartinLosi, AmandaWrześniewska-Wal, IwonaLu, WenyingWubishet, BefikaduLupi, Saturnino MarcoYang, Deng-HoMallio, Carlo A.Yoo, So YoungMarongiu, GiuseppeZang, LiqingMartins-Bessa, AnaZhang, GuangjuMassaro, SebastianoZhou, JonMatarese, GiovanniZhou, MingchuanMatsuzaki, ShinyaZielinski, TomaszMatthiesen, RuneZolotukhin, Igor A.


